# Study protocol. The Childhood Health, Activity, and Motor Performance School Study Denmark (The CHAMPS-study DK)

**DOI:** 10.1186/1471-2431-12-128

**Published:** 2012-08-20

**Authors:** Niels Wedderkopp, Eva Jespersen, Claudia Franz, Heidi Klakk, Malene Heidemann, Christina Christiansen, Niels Christian Møller, Charlotte Leboeuf-Yde

**Affiliations:** 1 Centre of Research in Childhood Health, Institute of Sports Science and Clinical Biomechanics, University of Southern Denmark, Campusvej 55, DK-5230, Odense M, Denmark; 2 Spine Center of Southern Denmark, SLB-Middelfart, Østre Houg vej 5, 5500, Middelfart, Denmark; 3 University College Lillebaelt, Odense, Denmark; 4 Peadiatric Research Center, The Hans Christian Andersen Children’s Hospital, Odense University Hospital, Kløvervænget, 5000, Odense C, Denmark; 5 Institute of Regional Health Service Research, University of Southern Denmark, Winsloewparken 19, 5000, Odense C, Denmark

## Abstract

**Background:**

An increasingly passive life-style in the Western World has led to a rise in life-style related disorders. This is a major concern for all segments of society. The county council of the municipality of Svendborg in Denmark, created six Sport Schools with increased levels of suitable physical activities, which made it possible to study the health outcomes in these children whilst comparing them to children who attended the ‘normal’ schools of the region using the design of a “natural experiment”.

**Methods:**

Children from the age of 6 till the age of 10, who accepted to be included in the monitoring process, were surveyed at baseline with questionnaires, physical examinations and physical and biological testing, including DXA scans. The physical examination and testing was repeated during the early stage of the study. Every week over the whole study period, the children will be followed with an automated mobile phone text message (SMS-Track) asking questions on their leisure time sports activities and the presence of any musculoskeletal problems. Children who report any such problems are monitored individually by health care personnel. Data are collected on demography, health habits and attitudes, physical characteristics, physical activity using accelerometers, motor performance, fitness, bone health, life-style disorders, injuries and musculoskeletal problems. Data collection will continue at least once a year until the children reach grade 9.

**Discussion:**

This project is embedded in a local community, which set up the intervention (The Sport Schools) and thereafter invited researchers to provide documentation and evaluation. Sport schools are well matched with the ‘normal’ schools, making comparisons between these suitable. However, subgroups that would be specifically targeted in lifestyle intervention studies (such as the definitely obese) could be relatively small. Therefore, results specific to minority groups may be diluted. Nonetheless, the many rigorously collected data will make it possible to study, for example, the general effect that different levels of physical activity may have on various health conditions and on proxy measures of life-style conditions. Specifically, it will help answer the question on whether increased physical activity in school has a positive effect on health in children.

## Background

An increasingly passive life-style in the Western World has led to a growth of life-style disorders already in childhood. This phenomenon has become a concern not only for the health care professions but also for the general public and hence for politicians and policy makers. In 2007, the county council of a municipality in Denmark, in the region of Svendborg, took the decision to provide increased levels of suitable physical activities in some of their primary schools, with the aim to improve the physical health of children; the Svendborg Project. This is a description of the Svendborg Project and the CHAMPS-study DK, which was made responsible for evaluating the project.

### The Svendborg project

The organization ‘Sport Study Svendborg’ brought forth the original concept and, thereafter, they became responsible for setting up the Svendborg project. All 19 primary schools in the area were invited to participate in the project, of which 6 agreed to partake. Their school leaders and physical education teachers were invited to design the set-up for an optimal sports school. Thus a committee consisting of people from these schools worked out a proposition based on Team Denmark’s age-related training concept [1]. According to this concept it is important to train children in a biologically relevant manner depending on their physical and physiological maturity. The final concept was accepted by the city council that also provided funding for 4 extra physical education lessons per week, which resulted in a minimum of 4.5 hrs per week, divided over at least 3 sessions per week and each session had to consist of at least 60 minutes. The norm in Denmark is 1.5 hours, corresponding to two sports lessons per week that may be reduced in effective time if they include changing of clothes and taking a shower afterwards.

Parents and children were unaware of the initiation of this project until two months before the following school year. At this time it was too late to ask for a change of school, meaning that parents could not select a sports school for their children at the detriment of an ordinary school or vice versa.

An advisory committee was thereafter established consisting of two central officers from the Kompan Company ( http://www.kompan.com), one of the world’s leading specialists in play solutions for all age groups, the Hospital Director from Odense University Hospital, the Director of Sport Study Svendborg, the Research Project Leader, and a secretary. The Advisory Committee meets once a year to advice on the politics and future activities of the Svendborg project, both for the Sport Schools and for the research.

A steering committee for the project was also established consisting of the schools’ coordinators, one from each participating school, the Director of Sport Study Svendborg, the Research Project Leader, and a secretary. The Steering Committee was responsible for the coordination of activities including research in and between schools.

### The CHAMPS-study DK

Eventually, the idea arose that it would be possible to evaluate the outcome of these activities, as there were six sports schools with an increased amount of physical activities and 13 schools doing “business as usual”. A researcher (NW) was contacted to be responsible for the evaluation of the effects of the extra sport and physical education on motor performance and health. A research program was established named ‘The Childhood Health, Activity and Motor Performance School Study – Denmark’ (The CHAMPS-study DK). For this, a research group was established, with the purpose of studying various potentially beneficial aspects of increased physical activity. The municipality was asked to provide six matched schools and obtained four, making it possible to make a comparison between the two types of schools. Funding for the research activities was obtained from external bodies.

Some data have now been collected for the first part of the study, but the study is on-going. It is the purpose of this paper to describe the organization of the CHAMPS-study DK, the main research questions, and the main methods of data collection.

### Organization of the CHAMPS-study DK

The research activities are lead by a senior researcher (NW), who is responsible for the employment and supervision of a number of researchers. Presently these are nine PhD students, one research assistant and several senior researchers as collaborator. In addition, a number of research assistants have been employed to assist with the data collection. Several external supervisors are also involved with the PhD projects.

The Advisory Committee and the Steering Committee of the Svendborg Project were both involved also with the CHAMPS-study DK, and the Svendborg county council provided a co-coordinator, to ensure that there would be communication between the Svendborg Project and the CHAMPS-study. Meetings were scheduled to be held regularly once a month during the school year.

## Methods

### Design

The research study (the CHAMPS-study) can be described as a natural experiment, in which the intervention (the Svendborg project) is not undertaken for the purpose of research but where, nevertheless, the variations in exposure (i.e. the ‘sports’ schools vs. the ‘normal’ schools) and outcomes can be analyzed with the intent of making causal inferences [2].

### Ethics approval and ethical considerations

Ethics committee approval was obtained before the start of the project; ID S20080047, and registration in the Danish Data Protection Agency was made, as stipulated by Danish law J.nr. 2008-41-2240. Since this was not a randomized controlled clinical trial, the study is not suitable for registration in any of the clinical trials registers.

Schools were all informed vocally and in writing about the project. Also the children and their parents were all informed in writing. In addition they were all invited to meetings at the different schools, where information regarding the research projects was presented before the start of the research. All parents or guardians provided informed written consent for the family to participate in the study. In relation to children, all participation was voluntary; children could at any time withdraw from the whole project or from any part of it. None of the tests were in any way dangerous; no risk was associated with any tests used.

In Denmark, each citizen is given a unique personal social security identity number, to which their address is linked. A database on participation was constructed, where, in addition to the study identity numbers, the personal social security numbers are kept securely. Only if results indicate disease can the personal identity number database be accessed to make it possible quickly to contact parents. Another database contains anonymous results and the two data sets can be linked through the study identity number. Hence no person referable data are available in the main data set. In addition, all reporting of data will be anonymous.

Schools, parents and children are kept informed on the progress of the project once a year through a newsletter.

### Study aims and main objectives

The main aim of this study is to describe differences in development of health and motor performance over time in relation to type of school and other background variables. This will be achieved through a number of studies. Thus in some analyses base-line factors will be used to predict outcome as measured with prospective data. Other analyses will be based on the differences between outcomes that arise over time in the sport and normal schools.

The main areas of interest are

1. Issues of life-style diseases, namely obesity and other risk indicators for type II diabetes and cardiovascular disease

2. Bone health

3. Musculoskeletal problems, and

4. Motor performance.

### Data collection in brief

The study commenced in 2008, including children from preschool (age 5) till grade 4 (age 10), with the purpose of following them including their 9th grade. All were surveyed at baseline with questionnaires, physical examinations and physical testing. Some tests are performed regularly, others only once or a few times. In the first three years of the study some procedures were repeated twice yearly. In the following years testing is planned for once a year. In addition, all children were and are followed once a week with a parental automated text message (SMS-Track) questionnaire. In addition, children who report any sort of musculoskeletal problems via SMS-Track are monitored individually by health care personnel.

### Examples of research areas are

#### Descriptive studies of parents and children

• Demographic description of children and their parents

• Health habits and attitudes in children and their parents

• Physical characteristics, motor performance and fitness measures of children

• The prevalence, incidence and tracking of indicators for life-style diseases

• The prevalence, incidence and tracking of injuries and back problems in children

• The status and tracking of bone health in children

#### Predictor studies of children

• The influence of the demographic baseline profile on outcomes such lifestyle disease indicators, musculoskeletal injuries and problems, bone health and also on physical activity levels during the study period

• Physical activity in relation to indicators for lifestyle disease such as type II diabetes, metabolic syndrome, and cardiovascular disease

• Physical fitness as a predictor for indicators for lifestyle disease such as type II diabetes, metabolic syndrome, and cardiovascular disease

• The risk of musculoskeletal injuries and problems in children, in relation to overweight, hyper mobility, level and type of physical activity, leisure time sport and type of sport, motor performance and previous injury

• Physical activity, fitness and motor performance in relation to bone health

#### Differences in outcome between types of schools

• Level of physical activity, fitness and motor performance

• Indicators for lifestyle disease such as type II diabetes, metabolic syndrome, and cardiovascular disease

• Musculoskeletal injuries and problems including back problems

• Bone health.

### The main players

#### The schools

The six sport schools and the four ‘normal’ or control schools were matched, in a way such that the area of uptake of the control schools matched that of the sport schools. Matching was based on the size and the distribution of socio-economic groups inside the uptake areas. During the first three years all children and parents were ‘faithful’ to the research studies, as in the first years a total of five children changed over between the two types of schools. Thus very little risk of selection bias occurred during the first years of the study through movement from one type to another type of school. Thus all analyses will be conducted on an ’intention to treat basis’, thus minimizing the risk of selection bias.

#### The physical education teachers in the sports schools

The concept of age related training is under constant development and the sports schools allocate resources for the continued education of their physical education teachers. This continued education is a priority of the schools and the municipality. Thus all physical education teachers at the sport schools were further educated in the age-related training concept designed by TEAM Danmark [1]. In addition, every year they attend “brush up” courses on the concept to keep up to date with new knowledge. All new physical education-teachers are sent on the basic course.

#### The parents and children

**Figure 1:**
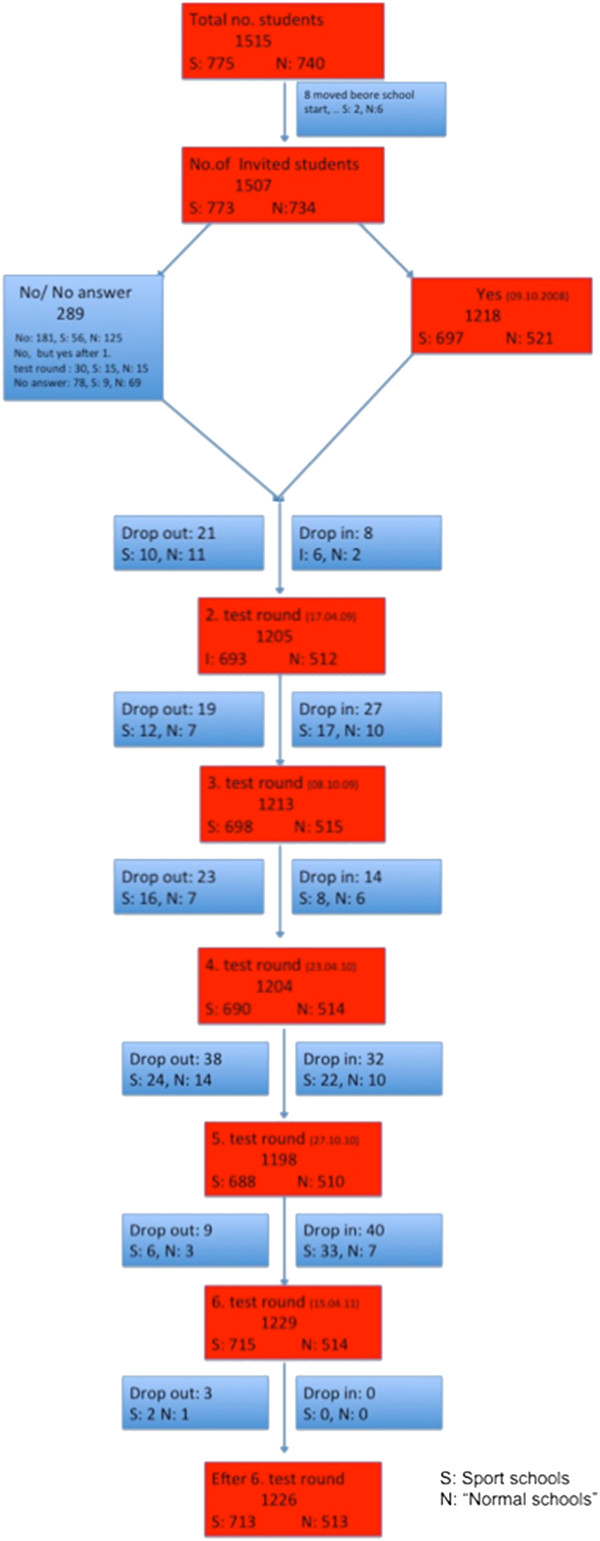
Flow chart of the CHAMPS-study DK, with Sport schools and normal schools.

All children and parents from kindergarten to 4th grade were asked to participate in the research program. In the sport schools 697 (90%) and in the control schools 521 (71%) agreed to participate. The study was kept open, so that new children could enter and leave the study at any time. The numbers of children entering and leaving the study the first three years are illustrated in the flowchart in Figure 1. Most children who left the study did so because they moved from the municipality. Only two children left the study because their parents disapproved of the study; feeling that the questions in the questionnaire were too private, in particular the issue of household income. Nearly the same number of children that left the study entered during the first three years. The study is on-going, and due to a new school structure, where children from smaller schools have been moved to larger schools and three new small schools have administratively been made part of three larger schools, the schools and the municipality have asked that these children get the same opportunity to participate also in the research program. Thus the study may in the near future increase with up to 600 children.

#### The testers

University students are either part of the research team (i.e. master’s and Ph.D. students) or employed to perform the physical tests. During each test round, 15–20 testers participate but not necessarily at the same each time. Prior to test rounds all these testers are trained by experienced researchers with a high degree of experience. The training is performed using children of the same ages as the test subjects at a school outside the municipality of Svendborg. During training, all procedures are performed several times to make sure that all testers are familiar with all tests.

#### Clinical personnel

A varying number of physiotherapists and chiropractors and one medical practitioner, all but one part of the research team (Ph.D. students), have been and are responsible for the follow-up of children who report pain or injuries. Every Monday they telephone parents whose children, according to the text message information, have had musculoskeletal problems in the past week If the problem still persists they will examine the child at his/her school within the next ten days. If there is a medical problem, the child is seen by the Research Leader, who is an orthopedic surgeon and Professor in Clinical Biomechanics. If needed, the child will be referred for para-clinical examination procedures.

#### The research team

The main research team (two senior researchers and a number of PhD students) is located at the Institute of Clinical Biomechanics and Sports Science and at the Institute of Regional Health Service Research. They all participate actively in all parts of the study. Other university students are involved in smaller aspects of the project from time to time.

### Main methods of data collection

#### Data collection methods

A number of data collection methods were and are used at different points in time (as described in Table 1. The methods of data collection included a questionnaire to the parents, physical examination and tests, the use of accelerometers, weekly text-message responses (SMS-Track), and – if necessary – a clinical examination. A subgroup of children was examined by whole body Dual energy X-ray Absorptiometry (DXA scan). For detailed information on some of the data collection methods, please refer to Additional file 1: Appendices 1 – 8.

**Table 1:** Summary of the data collection undertaken in the CHAMPS-study DK on school-children; frequency and method of data of collection and the variables collected

***FREQUENCY OF DATA COLLECTION***	***METHOD OF DATA COLLECTION***	***VARIABLES***
*At baseline*	*Questionnaire (App.1)*	·*Socio-economic-status*
		·*Previous habits and attitudes toward physical activity and diet*
		·*Demographics*
		·*Parental anthropometry, and more.*
*At baseline and twice a yr for 3 yrs, thereafter once a year*	*Physical examination (App.2)*	*Anthropometry of child:*
		·*Height*
		·*Weight*
		·*Waist circumference*
*At base-line and once a year*	*Physical examination (App.2)*	*Blood pressure*
*At baseline and once a year*	*Self-reported Tanner stage in structured interview(App.3)*	*Pubertal stage*
*At baseline and once a year*	*Physical examination (App. 4 and 5)*	·*Aerobic capacity*
		·*Motor performance*
		·*Hypermobility(according to Beghton’s criteria)*[3]
*Every second year*	*MTI GTX3 accelerometer (App.6)*	*Physical activity*
*Every second year*	*DXA scan (App.7)*	·*Bone mineral content*
		·*Bone mineral density*
		·*Bone area*
		·*Body composition*
*Weekly except during Christmas and summer holidays*	*SMS-Track (automated text messaging)(App.8)*	·*Musculoskeletal pain*
		·*Leisure time sports participation*
*When child reports pain in answer on text message question*	*Telephone consultation and clinical examination*	*Musculoskeletal complaints*
*When required*	*Clinical examination*	·*Continued back pain*
		·*Musculoskeletal pain and injuries*

### Statistical considerations

All data are entered twice and thereafter checked manually for outliers. However, the SMS-Track data are checked and inappropriate answers are cleaned immediately every week, as data are entered directly into the database from the text-message soft-ware program.

Presently, it is planned to perform analyses for three points of measurements of objectively measured physical activity, blood samples and DEXA scans and eight points for weight, height and aerobic and physical fitness. In addition, sport participation outside school will have been measured each week using mobile texting (SMS-track) during the school year for five years, amounting to 210 measurements of sports participation and musculoskeletal symptoms. Different types of statistical analyses will be performed based on research questions and type of data. For our analyses, a statistical power of 0.9 and a significance level of 0.05 have been chosen throughout the study. In relation to the required sample size, a number of considerations were necessary, as described below.

#### Considerations in relation to clusters within the study sample

The study is school-based and it is therefore possible that there are more differences in students between schools and classes than within individual schools and classes (cluster effect). This means that special statistical methods are needed that take this into account. For the various measurements they require the number of children in each group to be multiplied by a cluster effect of 3. This decision was based on findings in a previous study (the European Youth Heart study).

#### Sample size calculations for repeated measurements

A sample size calculation for repeated measurements on the measurement with the highest standard deviation (SD) and lowest number of repeated measurements was performed. Objectively measured physical activity (accelerometer data) has the highest SD and, with the assumption of a change of 50 counts per minute, a standard deviation of 200. With three measurements on each subject, 88 subjects in each group (a total of 176 subjects) are needed to show a difference without taking the cluster effect into account. With an assumed cluster factor of 3, 528 subjects will be needed for these analyses. A sample size of 1224 will make it possible to detect changes of less than 2% in blood pressure, fitness and body composition. Further, a change of less than 10% of the number of overweight children will be detectable, with this number of participants.

#### Multilevel analyses

Descriptive and simple bivariate analyses will be performed to describe the spread of data and to establish simple associations between potential predictor variables and the relevant outcome variables. In addition, multilevel multivariate analysis will be performed. In multivariate analysis, 50 participants need to be included for the first independent variable and eight for each of the subsequent variables [4]. With up to 25 independent variables, a ’cluster’ effect of 3, and a projected drop-out rate of 10%, it requires a minimum of 664 participants in the study. Thus with the participation at the moment, where 1224 children and teenagers participate and have participated for three years, a quite high power of the study is evident.

## Discussion

This study has several strong points. From an ethical point of view, this general intervention means that is that no children, such as the obese, are stigmatized, as all children are “treated”, regardless their physical state. Positive aspects, from a researcher’s point of view, are that it has many participants, making it possible to analyze a relatively large number of variables and covariates in multivariate analyses without exhausting the dataset. The study runs over a long period, which makes it possible to study the long-term course of life-style and life-style conditions. Specifically, it will answer some of the most important questions in the prevention of future life style diseases, such as type II diabetes and cardio vascular disease, such as: “Is it possible to change risk factor levels in children and adolescents through public intervention in schools?”, “Does additional physical education at school result in an overall increased physical activity?” and “Might increased quality of the physical activity, with activity tailored to the biological age of the children, have an effect on outcome?”

Further, it is the first study to include momentary assessment data (SMS-track) of children’s level of physical activity and musculoskeletal complaints and injuries. As these SMS data have a very high participation rate (>93% every week) over several years, it will be possible for the first time to study not only the incidence of musculoskeletal problems in children, but also the age of onset and the course over the years.

In addition, other state of the art measurements (DXA scans to measure body composition and accelerometers to measure physical activity measurements) together with high quality physical measurements with some of the best available methods for epidemiological studies are performed regularly. This together with very the high participation rates in all measurements make the study unique. The study will therefore provide credible new information on many aspects concerning childhood and adolescent health, and the implications for the future health in adulthood.

The study is also unique because it is a “study of a natural evolution” in a community introducing an intervention independently of researchers, a so-called natural experiment [2]. The role of the researcher is to measure the effect of the change introduced into the community but not to set up the experiment. It is community-based, meaning that its heterogeneity reflects that of the general population and not some possibly highly selected study sample. This heterogeneity requires a large number of participants, which fortunately was obtained in this study.

However, a potential problem with this is that interesting subgroups in the general population may be relatively small, such as the obese, as opposed to intervention performed by researchers, where the inclusion to the study would require a certain weight. Any effect of the extra physical intervention on such small subgroups may therefore be diluted on a group level, if the effect is only slight or none in children of normal weight.

Another potential limitation is that, although the two types of schools are well matched, making comparisons between them credible, and although the parental demographic profile of participants resembles that of its target population, some generalizability of the data may not extend beyond that type of population (small town with surrounding rural district in Denmark).

Another issue that needs careful consideration is the interpretation of data. When many analyses are performed, even if the study sample is large, spurious results are likely to occur. Therefore, unusual, single, or unexpected results must be carefully considered in relation to the profile of other results and their plausibility.

Nonetheless, the results obtained in this study are likely to have an impact on public health in relation to life-style related disorders in early life.

## Competing interests

None of the authors have any competing interests regarding the CHAMPS-study DK or this manuscript describing the protocol for the study, and the study has not received any funding or assistance from a commercial organization.

## Authors’ contributions

NW conceived the overall idea and started The CHAMPS-study DK, he wrote the draft of the manuscript and included the contributions from the other authors into the manuscript, he is supervising the data gathering and the clinical examinations of the children and he performs clinical examinations on all children who need to been seen in the secondary sector of the health system. EJ contributed scientifically to the study on injuries in childhood and youth, she is acquiring data on childhood and youth injuries and complaints, she has been critically revising the manuscript and has given final approval. CF contributed scientifically to the study on back problems in childhood and youth, she is acquiring data on childhood and youth back problems and complaints, she has been critically revising the manuscript and has given final approval. HK contributed scientifically to the study on risk factors of life style diseases, she has collected data the first three years and has supervised the testing procedures up to now, and taught master and bachelor students to test children, she has been critically revising the manuscript and given final approval. MH contributed scientifically to the study protocol on bone health in childhood and youth, she has acquired data and is planning for the third round of DXA-scans next year, she has been critically revising the manuscript and given final approval of the manuscript, CC contributed scientifically to the study protocol on motor performance and injuries in childhood and youth, she acquired data on childhood and injuries and motor performance, she has been critically revising the manuscript and given final approval of the manuscript, NCM contributed scientifically to the protocol on the study of physical activity and fitness in childhood and youth through the scientific knowledge and use of the MTI-accelerometer, he acquired data on childhood and youth activity, he has been critically revising the manuscript and given final approval, CLY has contributed scientifically to the study protocol on back problems and injury in childhood and youth, she has been critically revising the manuscript and given final approval. All authors read and approved the final manuscript.

## Pre-publication history

The pre-publication history for this paper can be accessed here:

http://www.biomedcentral.com/1471-2431/12/128/prepub

## Supplementary Material

Additional file 1 **Appendix 1.** Questionnaire regarding parents and their offspring. **Appendix 2.** Anthropometric measurements, blood pressure and physical activity. **Appendix 3.** Pubertal Stage. **Appendix 4.** Aerobic capacity. **Appendix 5.** Motor performance. **Appendix 6.** Physical activity measured with accelerometer. **Appendix 7.** Bone health measured with Dual Energy X ray Absorptiometry. **Appendix 8.** Musculoskeletal problems and leisure time sport participation collected with SMS Track [5-19]. Click here for file
