# Supplementary material for: Study protocol. The Childhood Health, Activity, and Motor Performance School Study Denmark (The CHAMPS-study DK)
Source: BMC Pediatr. 2012 Aug 20;12:128. doi: 10.1186/1471-2431-12-128 (PMC3483192; doi:10.1186/1471-2431-12-128)
Supplement: Additional file 1 — Appendix 1. Questionnaire regarding parents and their offspring. Appendix 2. Anthropometric measurements, blood pressure and physical activity. Appendix 3. Pubertal Stage. Appendix 4. Aerobic capacity. Appendix 5. Motor performance. Appendix 6. Physical activity measured with accelerometer. Appendix 7. Bone health measured with Dual Energy X ray Absorptiometry. Appendix 8. Musculoskeletal problems and leisure time sport participation collected with SMS Track [5-19]. [file 1471-2431-12-128-S1.pdf]

Dear Emily Crow

Thank you for your fast reply to our submission. All of our applications for money have been to non-profit foundations.

1. Can you please confirm whether your study protocol was reviewed by one of the funding bodies mentioned in your acknowledgements section? If a study has not been reviewed for scientific merit by the funding body, then the study protocol will be sent for peer-review with a member of our Editorial Board. If a study has received funding/assistance from a commercial organization, this should be clearly stated in the 'competing interests' section of your manuscript, and the study protocol will be sent for peer-review by a member of our Editorial Board.
   Please send proof of funding to the email address listed below. Proof of external funding should include details of the amount of funding that was awarded for your study. If your documents are not in English, please could you provide translated versions of the relevant parts. These should be endorsed and signed by a contactable person at the institution. Please also include the original documents.

The TRYG-foundation (http://trygfonden.dk/Om-TrygFonden/In-English) has donated 700.000 US $ as part of a large donation to The Center for Research in Childhood Health (http://www.sdu.dk/en/om_sdu/institutter_centre/rich). All donations for research from The TRYG-foudation goes through scientific review. In addition we received 150.000 US $ from The Research Foundation of Southern Denmark. The Research Foundation of Southern Denmark is a public foundation where all research applications are peer reviewed to make sure it is the best research projects that are funded. The Egmont Foundation donated 100.000 US $ as a part of a larger research donation to the Hans Christian Anders Childrens Hospital, Odense University hospital.
The following additional foudations have provided funding
Nordea Foundation 950.000 US $, The IMK foundation has provided 150.000 US $, The A.J. Andersen Foundation 10.000 US $, The Danish Rheumatism Association 10.000 US $, Østifternes Foundation 35.000 US $, Brd. Hartmanns Foundation 10.000 US $, TEAM Denmark Reseach Funds 250.000 US $, University College Lillebaelt 200.000 US $, University of Southern Denmark 600.000 US $, The Danish Chiropractor Foundation 250.000 US $, The Nordic Institute of Chiropractic and Clinical Biomechanics a non-govermental research institute provided office facilities. The Svendborg Project, Sport Study Svendborg, The Municipality of Svendborg provided the funding of the extra physical education on the sport schools and the coordination of the cooperation between schools and researchers.

I am attaching the documents for most of the direct donations, and hope this is OK, as it several administrations (university and different hospitals) that have the documents, and it has been difficult to get hold of these documents. If you would like the remaining, please let me know and I will get a hold of them. The administrative head of

The Center for Research in Childhood Health have endorsed this letter and the documents, which are all attached. He is available on e-mail: kfroberg@health.sdu.dk or by phone: +45 65503457, mobile phone: +4560113457.

2. Copy of the ethical approval is attached, there is two one for the first three years and one for shortly after prolonging the study.

3. One manuscript has been submitted to the Journal Osteoporosis. Title: Physical Activity at Different Intensities Affects Children's Bone Health. We expect to use BioMedCentral in the future. The above manuscript was in first place send to BMC Medicin, that rejected as they found the manuscript to not be of general interest.

4. The manuscript has been clarified, all parents or guardians did provide informed consent to participate in the study and all children provided verbal consent, this has been added to the manuscript. In the "Ethics approval and ethical considerations" section of the manuscript.

Kind regards

Niels Wedderkopp professor, ph.d., consultant
Orthopaedic dep. Hospital of Lillebaelt
Institute of Regional Health Service Research
Center of Research in Childhood Health,
University of Southern Denmark

Tlf. +45 6550 4499
Mobile: + 45
Email: nwedderkopp@health.sdu.dk
Addr. Campusvej 55, DK-5230 Odense M, Denmark

Karsten Froberg Associate Professor,
Head of Center of Research in Childhood Health,
Institute of Sports Science and Clinical Biomechanics.
University of Southern Denmark

Tel.+45 6550 3457
Mobile+45 6011 3457
Fax +45 6550 3480
Email: kfroberg@health.sdu.dk
Web: http://www.sdu.dk/ansat/kfroberg
Addr. Campusvej 55, DK-5230 Odense M, Denmark
